# Air Pollution and Stillbirth: A Population-Based Case–Control Study in Taiwan

**DOI:** 10.1289/ehp.1003056

**Published:** 2011-03-29

**Authors:** Bing-Fang Hwang, Yungling Leo Lee, Jouni J.K. Jaakkola

**Affiliations:** 1 Department of Occupational Safety and Health, College of Public Health, China Medical University, Taichung, Taiwan; 2 Institute of Epidemiology and Preventive Medicine and Research Center for Genes, Environment and Human Health, College of Public Health, National Taiwan University, Taipei, Taiwan; 3 Center for Environmental and Respiratory Health Research, Institute of Health Sciences, University of Oulu, Oulu, Finland; 4 Institute of Occupational and Environmental Medicine, University of Birmingham, Birmingham, United Kingdom

**Keywords:** air pollution, particle, stillbirth, sulfur dioxide

## Abstract

Background: There is limited evidence suggesting that prenatal exposure to ambient air pollutants may increase the risk of stillbirth, but previous epidemiological studies have not elaborated the most susceptible gestational period for the effects of air pollution exposure on stillbirth.

Objectives: We estimated associations between exposure to ambient air pollutants and stillbirth, with special reference to the assessment of gestational periods when the fetus is most susceptible.

Methods: We conducted a population-based case–control study in Taiwan. The case group consisted of 9,325 stillbirths, and the control group included 93,250 births randomly selected from 1,510,064 Taiwanese singleton newborns in 2001–2007. Adjusted logistic regression models were used to estimate odds ratios (ORs) per 10-ppb change for ozone and nitrogen dioxide, 1-ppb change for sulfur dioxide (SO_2_), 10-μg/m^3^ change for particulate matter with aerodynamic diameter ≤ 10 μm (PM_10_), and 100-ppb change for carbon monoxide during different gestational periods and according to term or preterm (< 37 weeks) birth status.

Results: Stillbirth increased in association with a 1-ppb increase in first-trimester SO_2_ [adjusted OR = 1.02; 95% confidence interval (CI), 1.00–1.04], particularly among preterm births (adjusted OR = 1.04; 95% CI, 1.01–1.07). Stillbirth was also associated with a 10-μg/m^3^ increase in PM_10_ during the first (adjusted OR = 1.02; 95% CI, 1.00–1.05) and second (adjusted OR = 1.02; 95% CI, 1.00–1.04) month of gestation, and, as with SO_2_, associations appeared to be restricted to preterm births (first-trimester adjusted OR = 1.03; 95% CI, 1.00–1.07).

Conclusion: The study provides evidence that exposure to outdoor air SO_2_ and PM_10_ may increase the risk of stillbirth, especially among preterm births, and that the most susceptible time periods for exposure are during the first trimester of gestation.

Epidemiologic studies since the 1990s have provided evidence that prenatal exposure to ambient air pollution may increase the risk of low birth weight and small for gestational age and preterm births (Lacasana et al. 2005; Maisonet et al. 2004; Ritz and Yu 1999; Ritz et al. 2007). There is limited evidence suggesting that prenatal exposure to ambient air pollutants may increase the risk of stillbirth, and previous studies have not identified which gestational periods are most susceptible to effects of air pollution on risks of stillbirth (Bobak and Leon 1999; Pearce et al. 2010; Pereira et al. 1998; Sakai 1984). In a Japanese ecological study, the risk for stillbirth was related to regional levels of nitrogen dioxide (NO_2_) and sulfur dioxide (SO_2_) (Sakai 1984). Another ecological study conducted in the Czech Republic reported an elevated, significant effect estimate of stillbirth for nitrogen oxides (NO_x_) but no association between SO_2_ and suspended particulate matter (PM) (Bobak and Leon 1999). A time-series study in Brazil reported a statistically significant association between daily exposure to NO_x_, SO_2_, and carbon monoxide (CO) and the risk of stillbirth (Pereira et al. 1998). A recent study conducted in northern England found no association between black smoke air pollution during pregnancy and the risk of stillbirth (Pearce et al. 2010).

We conducted a nationwide population-based case–control study in Taiwan to assess the effects of ambient air pollution exposure during pregnancy on the risk of stillbirth, with special reference to the assessment gestational periods when the fetus is most susceptible. These susceptible time windows of exposure could elucidate possible mechanisms underlying the effects of ambient air pollution on the risk of stillbirth. We focused on predominantly traffic-related pollutants [NO_2_, CO, and ozone (O_3_)] and air pollutants mainly from other fossil fuel combustion sources [SO_2_ and PM with aerodynamic diameter ≤ 10 μm (PM_10_)].

## Methods

*Study design.* We conducted a population-based case–control study based on a source population comprising 1,510,064 singleton births registered by the Taiwanese Birth Registry from 1 January 2001 through 31 December 2007.

All births are compulsorily reported to the Taiwan Birth Registry within 15 days of delivery. Taiwanese pregnant women are 99% covered by national health insurance, access to prenatal care is free of charge, and there are at least 10 prenatal care visits during pregnancy. A validation study of the Taiwanese birth registration data reported a low percentage of missing information (1.6%) and good validity (sensitivity and specificity were 92.8% and 99.6%, respectively) and reliability (Cohen’s *k*-statistic was 0.92) for preterm birth (< 37 weeks of gestational age) (Lin et al. 2004).

We identified all singleton stillbirths without any birth defects (*International Classification of Diseases, 9th Revision, Clinical Modification* codes 740–758) (Kase and Visintainer 2007) from the Taiwanese Birth Registry from 2001 to 2007. Birth defects and other health conditions are diagnosed mostly by pediatricians, and follow-up continues through 7 days after birth. The gestational age was based on ultrasound examination. We included stillbirths after 20 weeks of gestational age and excluded 30 stillbirths due to maternal smoking and 21 stillbirths from townships without air pollution monitoring data, leaving a total of 9,325 stillbirths. The controls were 93,250 births randomly selected (10 controls per case) from the source population of singleton births without birth defects or maternal smoking during pregnancy (680 births were excluded due to maternal smoking), excluding births from 25 of 365 townships located in the mountain area where there are no air monitoring stations.

*Exposure assessment.* Ambient air monitoring data for SO_2_, NO_2_, O_3_, CO, and PM_10_ are available for 72 Taiwan Environmental Protection Administration (EPA) monitoring stations on Taiwan’s main island since 1994. Concentrations of each pollutant are measured continuously—CO by nondispersive infrared absorption, NO_2_ by chemiluminescence, O_3_ by ultraviolet absorption, SO_2_ by ultraviolet fluorescence, and PM_10_ by beta-gauge—and are reported hourly.

We identified the map coordinates of the monitoring stations and air pollution sources. The data were managed by a geographic information system (ArcGIS version10; ESRI, Redlands, CA, USA). The air pollutant measurements from Taiwan EPA monitoring stations were integrated into monthly point data and interpolated to pollutant surfaces using inverse distance weighting (IDW). The monitoring data were assigned to individual women at postal-code area level. The postal code typically corresponded to one block face in urban areas but was larger in rural areas with low population density. This method provided high temporal resolution (daily measures for most days) and suitable spatial resolution (100 m) (Stroh et al. 2007). The air pollutant measurements from the three closest monitoring stations within 25 km of each residence were integrated for a monthly average for each woman during pregnancy. The details of the approach are described elsewhere (Hwang and Jaakkola 2008). We also performed sensitivity analyses focusing on the monitoring station closest to the postal code area of interest within 1 km (nearest method). The air pollution estimates were similar based on the nearest monitor and IDW methods (correlation coefficients > 0.87); therefore, we report estimates based on IDW methods only.

We calculated exposure parameters from the monthly 24-hr NO_2_, CO, SO_2_, and PM_10_ and 8-hr O_3_ average concentrations for the duration of pregnancies between 2000 and 2007. Based on the date of birth and gestational age, we estimated the monthly average concentration corresponding to the 9 months of gestation. We also estimated average concentration for the entire pregnancy, as well as for each trimester.

*Covariates.* Maternal age, sex of the infant, and season of conception (spring, summer, fall, winter) were routinely available from the birth registry. Municipal-level data from the Directorate-general of Budget, Account, Statistics, Executive Yuan, were used to determine municipal-level socioeconomic status (SES) based on the distribution of annual average incomes of households: high (> 75th percentile), medium (75th to 25th percentile), or low (< 25th percentile) SES.

*Statistical methods.* We explored the associations of interest for each month of pregnancy, each trimester, and the whole pregnancy to elaborate the relevant gestational period for stillbirth. We first applied logistic regression models including the covariates listed above to identify covariates associated with stillbirth and with air pollution concentrations, which were subsequently included in models investigating the association between air pollution exposures and stillbirth. Further, we also selected potential confounders *a priori* and included them into the final model.

Second, we fitted one-pollutant models, and then considered two-pollutant models by fitting one traffic-related and one stationary fossil fuel combustion-related pollutant. Finally, we fitted two-pollutant models with O_3_ and another pollutant. The two-pollutant models provide estimates of the independent effects of CO, NO_2_, SO_2_, PM_10_, and O_3_ on stillbirth while controlling for the second pollutant in the model. We also considered three-pollutant models with one traffic-related pollutant, one stationary fossil fuel combustion-related pollutant, and O_3_. The effect of each pollutant on the risk of stillbirth was estimated as odds ratios (ORs) per 10-ppb change for NO_2_ and O_3_, 1-ppb change for SO_2_, 100-ppb change for CO, and 10-μg/m^3^ change for PM_10_, along with their 95% confidence intervals (CIs). We further performed sensitivity analyses by comparing the effect estimates among preterm births (gestational age < 37 weeks) and term births (gestational age ≥ 37 weeks). Statistical significance was set at *p* < 0.05 based on a two-sided calculation.

## Results

*Characteristics of control and case subjects.* Table 1 presents characteristics of the study population. A larger proportion of cases than controls had younger (maternal age < 20 years) and older mothers (≥ 35 years). The mean of gestational age of cases was significantly lower than controls. Only 13% of stillbirths were born ≥ 37 weeks; most births during the first trimester were stillbirths. The distribution of cases over the birth year was different from the distribution of controls. We adjusted for these factors in the multivariate analysis.

**Table 1:** Distribution of characteristics [*n*(%)] among case and control subjects in a study of air pollution and stillbirth in Taiwan, 2001–2007.

Characteristic	Case subjects	Control subjects	*p*-Value
Total		9,325 (100)		93,250 (100)		
Preterm births		8,110 (87)		6,496 (7)		
Term births		1,215 (13)		86,754 (93)		
Gestational age at birth [weeks (mean ± SD)]		26.9 ± 6.3		38.5 ± 1.6		< 0.001
Maternal age (years)						< 0.001
< 20		652 (7.0)		3,147 (3.4)		
20–34		7,142 (76.6)		80,615 (86.5)		
≥ 35		1,531 (16.4)		9,488 (10.1)		
Sex of infant						0.90
Male		4,888 (52.4)		48,817 (52.4)		
Female		4,437 (47.6)		44,433 (47.6)		
SES*a*						0.33
Low		1,314 (14.1)		13,621 (14.6)		
Medium		5,492 (58.9)		54,864 (58.8)		
High		2,519 (27.0)		24,765 (26.6)		
Season of conception						0.29
Spring		2,353 (25.2)		23,657 (25.4)		
Summer		2,235 (24.0)		22,303 (23.9)		
Fall		2,180 (23.4)		22,452 (24.1)		
Winter		2,557 (27.4)		24,838 (26.6)		
Year of birth						< 0.001
2001		1,341 (14.4)		14,861 (15.94)		
2002		1,252 (13.4)		14,693 (15.76)		
2003		1,321 (14.17)		13,344 (14.31)		
2004		1,355 (14.54)		13,036 (13.98)		
2005		1,356 (14.54)		12,491 (13.40)		
2006		1,362 (14.60)		12,386 (13.28)		
2007		1,338 (14.88)		12,439 (13.34)		
**a**Municipal-level SES based on the distribution of annual average incomes of households: high (> 75th percentile), medium (75th to 25th percentile), or low (< 25th percentile).						

*Air pollution.* Table 2 presents the distributions of the monthly mean air pollutant concentrations during the study period. The correlation during the 9 months of gestation was high between NO_2_ and CO average concentrations (*r* = 0.62), which represent the common source of motor vehicles (Table 3). The concentrations of PM_10_ and SO_2_ were also moderately correlated (*r* = 0.53), indicating a common source of stationary fuel combustion, although SO_2_ concentrations were also associated with both traffic-related pollutants. The concentration of O_3_ was negatively associated with the mainly traffic-related pollutants but positively with PM_10_ and SO_2_, and it was only weakly associated with that of traffic-related and stationary fossil-fuel combustion-related air pollutants (Table 3).

**Table 2:** Distributions of air pollution concentration estimates by IDW methods during the study period.

Air pollutant	Mean ± SD	Minimum	Maximum	Interquartile range
PM_10_ (μg/m^3^)		72.95 ± 23.33		34.05		125.86		35.18
SO_2_ (ppb)		5.74 ± 2.67		1.97		15.92		1.82
NO_2_ (ppb)		21.7 ± 7.93		3.47		40.08		9.74
CO (ppm)		0.66 ± 0.18		0.27		1.21		0.19
O_3_ (ppb)		35.93 ± 9.61		14.00		61.27		13.03

**Table 3:** Correlations of trimester average concentrations of main air pollutants during pregnancy.

Air pollutant	CO	NO_2_	O_3_	PM_10_	SO_2_
CO		1.00		0.62*		–0.45*		–0.10		0.15
No_2_				1.00		–0.31*		0.10		0.41*
O_3_						1.00		0.62*		0.13
PM_10_								1.00		0.53*
SO_2_										1.00
*p < 0.05.										

*Air pollution and stillbirth.* A 10-μg/m^3^ increase in PM_10_ during the first trimester was weakly but not significantly associated with stillbirth overall (adjusted OR = 1.02; 95% CI, 0.99–1.04). We found a significant association between first-trimester PM_10_ and stillbirth among preterm births (OR = 1.03; 95% CI, 1.00–1.07) but no evidence of an association among term births or of associations with PM_10_ exposures during other time periods (overall or among preterm or term births) (Table 4). Associations with PM_10_ during the first, second, and third months of gestation were similar to those for the first trimester as a whole, but ORs for the first 3 months among preterm births were slightly stronger when adjusted for O_3_ and either CO or NO_2_ (Table 5).

**Table 4:** Adjusted ORs (95% CIs) for stillbirth by average pollutant concentrations, by trimester and for the whole pregnancy: single-pollutant models.

Air pollutant	All births (gestational age > 20 weeks)*a*	Preterm births (gestational age < 37 weeks)*b*	Term births (gestational age ≥ 37 weeks)*b*
PM_10_ (10 μg/m^3^)						
First trimester		1.02 (0.99–1.04)		1.03 (1.00–1.07)		1.00 (0.96–1.04)
Second trimester		0.96 (0.94–0.99)		0.99 (0.95–1.02)		0.95 (0.91–0.99)
Third trimester		0.97 (0.94–1.00)		0.97 (0.92–1.02)		0.98 (0.94–1.01)
Whole pregnancy		0.98 (0.94–1.01)		1.00 (0.96–1.05)		0.96 (0.91–1.00)
SO_2_ (1 ppb)						
First trimester		1.02 (1.00–1.04)		1.04 (1.01–1.07)		1.00 (0.97–1.02)
Second trimester		1.00 (0.98–1.02)		1.00 (0.94–1.07)		0.99 (0.96–1.02)
Third trimester		1.00 (0.98–1.02)		1.01 (0.97–1.04)		0.99 (0.97–1.02)
Whole pregnancy		1.01 (0.99–1.03)		1.03 (1.00–1.06)		0.99 (0.96–1.02)
NO_2_ (10 ppb)						
First trimester		1.01 (0.96–1.07)		1.04 (0.97–1.12)		0.98 (0.90–1.06)
Second trimester		0.96 (0.92–1.02)		1.00 (0.93–1.07)		0.95 (0.88–1.02)
Third trimester		0.98 (0.92–1.04)		0.98 (0.89–1.08)		0.98 (0.91–1.06)
Whole pregnancy		0.98 (0.92–1.04)		1.02 (0.94–1.10)		0.96 (0.88–1.05)
CO (100 ppb)						
First trimester		1.00 (0.98–1.02)		0.99 (0.97–1.02)		1.01 (0.98–1.04)
Second trimester		0.99 (0.97–1.01)		0.98 (0.96–1.01)		1.01 (0.98–1.03)
Third trimester		1.01 (0.99–1.03)		0.98 (0.95–1.02)		1.02 (0.99–1.05)
Whole pregnancy		1.00 (0.98–1.02)		0.99 (0.96–1.01)		1.01 (0.98–1.04)
O_3_ (10 ppb)						
First trimester		1.01 (0.96–1.06)		1.02 (0.95–1.09)		0.99 (0.92–1.06)
Second trimester		0.96 (0.91–1.01)		1.00 (0.94–1.07)		0.91 (0.85–0.98)
Third trimester		0.98 (0.93–1.04)		0.98 (0.93–1.08)		0.99 (0.92–1.06)
Whole pregnancy		0.97 (0.91–1.04)		1.00 (0.91–1.09)		0.93 (0.85–1.03)
**a**Logistic regression analysis adjusting for sex, maternal age, gestational age, municipal-level SES, season of conception, and year of birth. **b**Logistic regression analysis adjusting for sex, maternal age, municipal-level SES, season of conception, and year of birth.						

**Table 5:** Adjusted ORs (95% CIs) for stillbirth by average concentrations of the first 3 months of gestation.

Single-pollutant model	Three-pollutant models						
	Air pollutant	SO_2_ + CO + O_3_	SO_2_ + NO_2_ + O_3_	PM_10_ + CO + O_3_	PM_10_ + NO_2_ + O_3_
All births*a*										
PM_10_ (10 μg/m^3^)										
First month		1.02 (1.00–1.05)						1.02 (0.99–1.06)		1.02 (0.99–1.06)
Second month		1.02 (1.00–1.04)						1.03 (1.00–1.06)		1.03 (1.00–1.07)
Third month		1.00 (0.98–1.03)						1.01 (0.98–1.04)		1.01 (0.98–1.05)
SO_2_ (1 ppb)										
First month		1.02 (1.00–1.04)		1.02 (1.00–1.04)		1.02 (1.00–1.04)				
Second month		1.02 (1.00–1.04)		1.02 (1.00–1.04)		1.02 (1.00–1.04)				
Third month		1.01 (1.00–1.03)		1.02 (1.00–1.03)		1.02 (1.00–1.04)				
Preterm births*b*										
PM_10_ (10 μg/m^3^)										
First month		1.03 (1.00–1.06)						1.04 (1.00–1.09)		1.04 (0.99–1.09)
Second month		1.03 (1.00–1.06)						1.05 (1.01–1.09)		1.05 (1.00–1.09)
Third month		1.02 (0.99–1.05)						1.03 (0.99–1.08)		1.04 (0.99–1.09)
SO_2_ (1 ppb)										
First month		1.04 (1.02–1.06)		1.04 (1.02–1.07)		1.05 (1.02–1.08)				
Second month		1.04 (1.01–1.06)		1.05 (1.02–1.07)		1.05 (1.02–1.09)				
Third month		1.03 (1.01–1.06)		1.04 (1.01–1.06)		1.05 (1.02–1.09)				
Term births*b*										
PM_10_ (10 μg/m^3^)										
First month		1.01 (0.97–1.04)						1.00 (0.96–1.05)		1.01 (0.96–1.06)
Second month		1.01 (0.97–1.04)						1.01 (0.96–1.05)		1.02 (0.97–1.07)
Third month		0.98 (0.95–1.02)						0.98 (0.94–1.02)		0.99 (0.94–1.04)
SO_2_ (1 ppb)										
First month		1.00 (0.98–1.03)		1.00 (0.97–1.03)		1.00 (0.97–1.03)				
Second month		1.00 (0.98–1.03)		1.00 (0.97–1.03)		1.00 (0.97–1.03)				
Third month		0.99 (0.97–1.02)		0.99 (0.97–1.02)		1.00 (0.97–1.03)				
**a**Logistic regression analysis adjusting for sex, maternal age, gestational age, municipal-level SES, season of conception, and year of birth. **b**Logistic regression analysis adjusting for sex, maternal age, municipal-level SES, season of conception, and year of birth.										

A 1-ppb increase in SO_2_ during the first trimester was significantly associated with stillbirth among all births (adjusted OR = 1.02; 95% CI, 1.00–1.04), but stratified estimates indicated that the association was present only among preterm births (adjusted OR = 1.04; 95% CI, 1.01–1.07) (Table 4). Associations with SO_2_ during the first, second, and third months of pregnancy were similar to those for the first trimester, and ORs for the first 3 months among preterm births were slightly stronger with adjustment for O_3_ and CO or NO_2_ (Table 5).

NO_2_, CO, and O_3_ exposures were not associated with stillbirth in the study cohort regardless of the timing of exposure or preterm versus term birth status (Table 4) or adjustment for other pollutants (data not shown).

## Discussion

In our large population-based case–control study, the risk of stillbirth increased in association with SO_2_ and PM_10_ levels during the first and second months of pregnancy. The effect estimate indicating an approximately 2% increase in the odds of stillbirth per 1-ppb increase in SO_2_ level was stable with adjustment for different combinations of air pollutants in the multipollutant models. However, the association with first-trimester SO_2_ appeared to be limited to preterm births, with a 4–5% increase in the odds of stillbirth per 1-ppb increase in SO_2_. PM_10_ exposure during the first and second month of pregnancy was associated with stillbirth overall (2–3% increased odds per 10-μg/m^3^ increase in PM_10_) and among preterm births specifically (4–5% increased odds). The results provide evidence that SO_2_ and PM_10_ exposure during early pregnancy may increase the risk of stillbirth.

*Validity of results.* We based our outcome of interest on birth registration. Because it is possible that birth defects may relate to other exposures that also may mediate the risk of stillbirth, we excluded all case and control subjects with any birth defect. We adjusted potential confounding by gestational age, maternal age, season of conception, and SES in the logistic regressions in all births. We eliminated potential confounding or effect modification by maternal smoking by focusing on newborns of nonsmoking mothers. From another study report (Shih et al. 2007), we know that the prevalence of smoking during pregnancy in Taiwan is much higher (4.0%) than the information in the records (0.3% among cases’ mothers and < 0.05% among controls). It could reflect underreporting information in the records, and therefore residual confounding could be an issue.

It was not possible to adjust for some potential confounders, including nutritional, behavioral, and other environmental and occupational factors (Yakoob et al. 2009), because no such information was available on Taiwanese birth registration. Because these factors may vary seasonally and have regional variation, we adjusted for season of conception and a municipal-level measure of SES. However, residual confounding is still possible by unmeasured or poorly characterized factors or by other environmental toxicants.

Any known or unknown factors, such as physical activity, time spent outdoors, occupational status, air exchange, penetration, deposition, or indoor pollutants could be responsible for the observed association between personal exposure and municipal-level exposure. A major strength of our population-based case–control study based on the Taiwan Birth Registry is the large number of births, which reduces uncertainty due to random error that is a concern for smaller studies that collect detailed information on covariates directly from pregnant women (Ritz and Wilhelm 2008).

We based our exposure assessment on residential postal code rather than on address during pregnancy, and we used a geographic information system to integrate monthly air pollutant data from 72 Taiwan EPA monitoring stations, which we interpolated to pollutant surfaces using IDW. A previous study reported that using municipal-level exposures obtained from air pollution monitoring stations as a proxy for personal exposure results in smaller effect estimates than estimates based on individual-level exposure (Navidi and Lurmann 1995). A plausible source of information bias is that pregnant women may change residential location, which will lead to exposure misclassification. Based on reports from the United States (13%), Canada (12%), and Australia (19%), a substantial proportion of the population moves during pregnancy (Chen et al. 2010; Fell et al. 2004; Raynes-Greenow et al. 2008), which may decrease the accuracy of exposure assessment and introduce nondifferential misclassification that would most likely result in underestimation of air pollution effects.

Although the validity of the Taiwanese registry for preterm birth is fairly good (Lin et al. 2004), stillbirths may be undercounted. PM_10_ was measured using a beta-gauge method that is sensitive to temperature and relative humidity, but we did not correct monitoring station measured values for these factors. We classified SES at the municipal level, which may not accurately capture individual SES in large cities.

*Synthesis with previous knowledge.* Four previous studies, conducted in Japan (Sakai 1984), Brazil (Pereira et al. 1998), Czech Republic (Bobak and Leon 1999), and northern England (Pearce et al. 2010), have investigated associations between exposure to ambient air pollution and stillbirth. The Japanese and Czech studies focusing on effects of annual exposure were semiecological; that is, the exposure assessment was based on regional air pollution levels. The Brazilian time-series study assessed the effects of daily exposure up to 14 days before stillbirth only. A retrospective cohort study conducted in northern England explored the association between stillbirth and exposure to PM during pregnancy. The present study estimates associations with exposure in 1-month time windows over the entire pregnancy. We found a 2% increase in the odds of stillbirth per 1-ppb increase in SO_2_ exposure during the first trimester of gestation. This is consistent with the results of the Japanese study of four districts (Sakai 1984), in which stillbirth was associated with the regional level of SO_2_, and with the Brazilian study (Pereira et al. 1998), which reported a nonsignificant association between SO_2_ exposure and intrauterine mortality (coefficient = 0.0038 per μg/m^3^; *p* < 0.1). The Japanese study estimated associations with average SO_2_ exposure concentration during pregnancy, whereas in the Brazilian study the effect estimate was presented for a 5-day moving average exposure. In an ecological study of 45 districts in the Czech Republic (Bobak and Leon 1999), the adjusted OR was reduced in relation to SO_2_ (OR = 0.90; 95% CI = 0.70–1.16 per 50 μg/m^3^), although not statistically significantly. In the present study stillbirth was also associated with a 10-μg/m^3^ increase in PM_10_ during the first and second month of gestation, which is consistent with the Brazilian study (OR = 1.01; 95% CI, 1.00–1.02 per 10 μg/m^3^ PM_10_) (Pereira et al. 1998). Further, we found a positive association between PM_10_ exposure in the first trimester and stillbirth among preterm births. A retrospective cohort study of 90,537 singleton births over 30 years (1962–1992) in a small city in the United Kingdom reported no association between stillbirth and PM exposure (measured as black smoke) during any trimester or the entire pregnancy (OR = 1.010; 95% CI = 0.991–1.028 per 10 μg/m^3^) (Pearce et al. 2010). An apparent difference between the United Kingdom and our study may result from different scales of measurement error and bias. We did not observe an association between CO exposure and stillbirth. The Brazilian study (coefficient = 0.0223 per 1 ppm; *p* < 0.1) reported a nonsignificant association between a 1-ppm increase in short-term CO exposure and the risk of stillbirth. Neither the Japanese nor Czech study investigated associations with CO (Bobak and Leon 1999; Sakai 1984). In the present study, we found a nonsignificant association between stillbirth and a 10-ppb increase in NO_2_ for each trimester and for the whole pregnancy. It is possible that some of our statistically significant findings could reflect chance, given the large number of estimates generated. Although our findings suggest that SO_2_ and PM_10_ exposures were associated with stillbirth, and that the most susceptible time window was during the first trimester, we cannot rule out the possibility of chance.

*Biological mechanisms.* It is not known how exposure to SO_2_ and PM_10_ in the early fetal period might contribute to stillbirth. A possible biological mechanism is that atmospheric sulfur oxides may increase methemoglobin levels, which interfere with the oxygen-carrying capacity of hemoglobin in children. Infants are more sensitive to such events because fetal hemoglobin is more likely to be oxidized to methemoglobin (Petr and Schmidt 1966). Maternal exposure to PM air pollutants during pregnancy can result in increased concentration of DNA adducts or decreased efficiency of the transplacental function with consequent deterioration in fetal growth and development of stillbirth in humans (Perera et al. 1992; Zondervan et al. 1987). It is not clear yet whether toxic components of PM_10_ or other measured (SO_2_, CO, NO_2_, and O_3_) or unmeasured compounds associated with PM_10_ (e.g., polycyclic aromatic hydrocarbons) might interfere with mechanisms regulating fetal growth and contribute to the etiology of stillbirth.

## Conclusions

The present study suggests that exposure to outdoor air SO_2_ and PM_10_ during the first trimester of pregnancy may increase the risk of stillbirth, particularly among preterm births.
